# Cancer in ANCA-Associated Glomerulonephritis: A Registry-Based Cohort Study

**DOI:** 10.1155/2017/6013038

**Published:** 2017-12-18

**Authors:** Sanjeevan Sriskandarajah, Leif Bostad, Tor Åge Myklebust, Bjørn Møller, Steinar Skrede, Rune Bjørneklett

**Affiliations:** ^1^Department of Clinical Medicine, University of Bergen, Bergen, Norway; ^2^Department of Pathology, Haukeland University Hospital, Bergen, Norway; ^3^Department of Clinical and Registry-Based Research, Cancer Registry of Norway, Institute of Population-Based Cancer Research, Oslo, Norway; ^4^Department of Clinical Science, University of Bergen, Bergen, Norway; ^5^Department of Medicine, Haukeland University Hospital, Bergen, Norway; ^6^Emergency Care Clinic, Haukeland University Hospital, Bergen, Norway

## Abstract

**Background:**

Immunosuppressive therapy for antineutrophil cytoplasmic antibody-associated vasculitis has been associated with increased malignancy risk.

**Objectives:**

To quantify the cancer risk associated with contemporary cyclophosphamide-sparing protocols.

**Methods:**

Patients from the Norwegian Kidney Biopsy Registry between 1988 and 2012 who had biopsy-verified pauci-immune glomerulonephritis and positive antineutrophil cytoplasmic antibody (ANCA) serology were included. Standardised incidence ratios (SIRs) were calculated to compare the study cohort with the general population.

**Results:**

The study cohort included 419 patients. During 3010 person-years, cancer developed in 41 patients (9.79%); the expected number of cancer cases was 37.5 (8.95%). The cohort had SIRs as follows: 1.09, all cancer types (95% CI, 0.81 to 1.49); 0.96, all types except nonmelanoma skin cancer (95% CI, 0.69 to 1.34); 3.40, nonmelanoma skin cancer (95% CI, 1.62 to 7.14); 3.52, hematologic cancer (95% CI, 1.32 to 9.37); 2.12, posttransplant cancer (95% CI, 1.01 to 4.44); and 1.53, during the 1–5-year follow-up after diagnosis (95% CI, 1.01 to 2.32).

**Conclusions:**

Cancer risk did not increase significantly in this cohort with ANCA-associated glomerulonephritis. However, increased risk of nonmelanoma skin cancer, posttransplant cancer, and hematologic cancer indicates an association between immunosuppression and malignancy.

## 1. Introduction

Historically, antineutrophil cytoplasmic antibody- (ANCA-) associated vasculitis (AAV) was a fatal disease. The introduction of cyclophosphamide (CYC) treatment in the 1960s improved the prognosis and made long-term survival possible for patients with AAV [1, 2]. However, evidence soon emerged that the long-term survival of patients with AAV was associated with significant morbidity including a substantially increased cancer risk [3–7]. Immunosuppressive therapy using CYC was particularly associated with malignancy. Although the severe adverse effects have elicited a search for less toxic treatment regimens, CYC still remains the first-line drug [8].

High occurrence of cancer has been demonstrated in patients with AAV treated with cumulative CYC doses exceeding 36 g [9–11] or when the treatment duration lasted for more than a year [4, 12–14]. In the current clinical practice, cumulative CYC doses and treatment duration rarely exceed these limits [15–18], which questions the relevance of previous CYC-associated increased cancer risk observations in patients with AAV. There is a relative paucity of data regarding cancer risk in AAV patients treated with current therapy protocols, and, to our knowledge, only 4 studies have published such data [5, 11, 13, 19]. In these studies, increased incidence of malignancy was observed. After excluding nonmelanoma skin cancer (NMSC) from the analyses, the incidence of malignancy no longer significantly increased in any of these investigations. However, these 4 studies were relatively small, with a limited statistical power to detect small- to medium-range associations between cancer and AAV. Furthermore, most of these reports are single-center studies, which limits their generalizability.

Interestingly, in the most recent study, the standardised incidence ratio (SIR) of all cancer types treated with CYC was 4.61 (95% confidence interval (CI) 1.16 to 39.38) times higher than that with rituximab-based therapy. However, after excluding NMSC from their analysis, the risk was only 1.30 (95% CI not reported) times higher with CYC compared with rituximab-based therapy [19].

To further investigate the association of cancer and AAV, we analysed data from the Norwegian Kidney Biopsy Registry (NKBR) and the Norwegian Cancer Registry. Additionally, we merged data from the present and the 4 recent studies on this topic and investigated the cancer risk in a total of 1532 patients with AAV diagnosed after 1988.

## 2. Materials and Methods

The study was approved by the Regional Committees for Medical and Health Research Ethics (REC South-East 2013/1083).

### 2.1. Study Population and Registries

The NKBR was established in 1988. We estimate that ~90% of all kidney biopsies are registered. The registry contains morphological, laboratory, and clinical data collected when the biopsy was performed. The Norwegian Cancer Registry was established in 1953. Reporting to this registry is mandatory and based on reports from clinical and pathological departments and death certificates. A near complete registration (98-99%) of solid tumors, except for basal skin cell carcinomas, is documented [20]. Annual sex-specific incidence rates for cancer and cancer subtypes are available for age groups and time periods in 5-year intervals. These data allow for an accurate calculation of the expected cancer case numbers in the study cohort. The Norwegian Cause of Death Registry is part of Statistics Norway and based on the mandatory Norwegian death certificate. The Norwegian Renal Registry was established in 1980 and has registered all patients with end-stage renal disease, defined by the commencement of maintenance dialysis or receiving kidney transplantation.

### 2.2. Data Collection and Definitions

We included patients registered in the NKBR and diagnosed from 1988 to 2012, with a pauci-immune necrotising glomerulonephritis and a positive ANCA serology. Patients with cancer prior to the AAV diagnosis were excluded. Baseline clinical data, including sex, age, ANCA specificity, and estimated glomerular filtration rate (eGFR) (determined by the Modification of Diet in Renal Disease equation) [21] were obtained from the NKBR. The primary study end-point was incidence of cancer. By using the unique 11-digit Norwegian personal number, the study cohort was linked with the Norwegian Cancer Registry to identify the cancer incidence. Causes of deaths in the study cohort were identified through linkage with the Norwegian Cause of Death Registry and classified as vascular, malignant, infectious, or active inflammation and other causes. The observation period was from the kidney biopsy date to incident cancer, end of 2013, or death, whichever came first. Patients with end-stage renal disease and those receiving kidney transplants were identified through record linkage with the Norwegian Renal Registry. When we calculated the cancer risk in transplanted patients, the observation period started from the kidney transplantation date.

### 2.3. SIR Calculation

We calculated the SIR as the ratio between the observed and expected cancer case numbers in the cohort. The expected cancer case number was calculated as follows: first, the number of person-years was calculated in the cohort, stratified by 5-year age groups and 1-year time periods. Second, this person-time was multiplied with the corresponding incidence rate in the general population to get the expected number of cases in each age group and 1-year period. The total number of expected cancer cases was then calculated as the sum of expected cases across age groups and time periods. The observed cancer case number was determined by record linkage of the study cohort and the Norwegian Cancer Registry using the 11-digit unique Norwegian personal number. A Poisson distribution of cancer incidence was assumed when 95% CIs were calculated. We calculated the SIR throughout the study period and stratified in accordance with disease duration periods (0-1, 1–5, 5–10, and >10 years after kidney biopsy), sex, ANCA specificity [cytoplasmic ANCA (C-ANCA)/proteinase 3 ANCA (PR3-ANCA) or perinuclear ANCA (P-ANCA)/myeloperoxidase ANCA (MPO-ANCA)], time periods (1988–2002 and 2003–2012), and posttransplantation observation period.

### 2.4. Pooled Analysis

A systematic PubMed search was conducted to identify previous studies reporting the cancer risk in patients with AAV diagnosed after 1988. The search was restricted to papers published in English language. Studies who do not report SIR data were excluded. The SIRs were calculated as the sum of the observed cancer cases divided by that of the expected cancer cases in all studies.

### 2.5. Treatment

Information regarding cumulative CYC doses administered to the patients is not available in the NKBR. In a previous Norwegian study including patients with Wegener's granulomatosis diagnosed between 1988 and 1998, the majority of patients received intravenous CYC with a median cumulative dose of 17 g. In patients receiving oral CYC, the median cumulative dose was 48 g [22]. Most centers since approximately 2003 have substituted CYC with azathioprine for maintenance treatment, substantially lowering the exposure to CYC [23]. In some patients, rituximab has been used for induction and maintenance treatment. Some patients have also received plasma exchange treatments [24].

### 2.6. Statistical Analyses

Continuous variables were expressed as medians with 25th and 75th percentiles and categorical variables as numbers (%). Comparisons of continuous and categorical variables in the baseline characteristics were calculated using the Mann–Whitney* U* test and the *X*^2^ or Fisher's exact test, respectively. A two-tailed *p* value of ≤0.05 and 95% CI was considered statistically significant. All statistical analyses were performed using the SPSS software, V.23, and STATA software, V.14.

## 3. Results

### 3.1. Baseline Characteristics

Between 1988 and 2012, 454 patients diagnosed with AAV and glomerulonephritis were identified. Of these, 35 were excluded as a result of cancer diagnosis prior to the observation period. Thus, 419 patients were included in the study cohort. As shown in Table 1, the median age in the cohort was 62 years [interquartile range, 48 to 72 years], and 229 (55%) were men. The median eGFR at the time of kidney biopsy was 23 mL/min/1.73 m^2^ (interquartile range, 11 to 46). A positive C-ANCA/PR3-ANCA was found in 237 (57%) and P-ANCA/MPO-ANCA in 183 (43%) patients.

The median length of follow-up was 5.7 years (interquartile range, 2.8 to 11.3). The mean length of follow-up was 7.2 years (standard deviation, 5.8), and the total number of person-years of observation was 3010. A total of 148 (35%) patients died during the observation period. Causes of deaths are registered for 138 patients, of which 69 (50%) died from infectious disease or active inflammation/vasculitis, 36 (26%) cardiovascular disease, 12 (9%) malignancy, and 21 (15%) from other causes. Kidney transplantation was performed in 60 (14%) patients.

### 3.2. Observed Cancer Cases

During follow-up, 46 cancer cases were reported in 41 (9.5%) patients. The first occurring cancer cases were as follows: NMSC (7 cases), lung (7), prostate (5), hematologic (4), urinary bladder and ureter (3), rectum (3), colon (2), uterus (2), central nerve system (2), unknown primary site (2), thyroid (1), ovary (1), lymphoma (1), and pancreas (1). Five patients had 2 distinct cancer diagnoses; the second occurring cancer cases were as follows: lung (1), NMSC (1), prostate (1), hematologic (1), and stomach (1). Seven patients who received transplants were diagnosed with cancer; the first occurring cancer cases were as follows: lung (4), prostate (1), lymphoma (1), and NMSC (1). Two of the 5 patients with 2 distinct cancers underwent transplant, with the cases of secondary cancer involving the prostate and lungs.

### 3.3. Comparison of Patients with and without Cancer

The comparison of the patients with and without cancer during follow-up is shown in Table 1. The patients with cancer were significantly older [65 years versus 61 years (*p* = 0.04)] at the time of AAV diagnosis, and a significantly higher percentage were men (71% versus 53% (*p* = 0.03)). There were no significant differences in eGFR and ANCA specificity between those with cancer and those without cancer during follow-up. A higher percentage of patients with cancer died during follow-up [25 (61%) versus 123 (33%) (*p* < 0.001)].

### 3.4. Comparison with the General Population

As shown in Table 2(a), the SIR of overall malignancy was 1.09 (95% CI 0.81 to 1.49). The SIR of all cancer types, except NMSC, was 0.96 (95% CI 0.69 to 1.34). In the 1–5-year period after AAV diagnosis, the cancer risk in the study cohort significantly increased compared to that in the general population (SIR, 1.53; 95% CI 1.01 to 2.32). The SIR did not increase in the first year or >5 years of follow-up after AAV diagnosis. In gender-, ANCA-, and time period specificity-stratified analyses, SIR of cancer was not significantly increased for males of 1.27 (95% CI 0.88 to 1.83), in the C-ANCA/PR3-ANCA positive group of 1.17 (95% CI 0.78 to 1.74) and in the 1988–2002 time period of 1.14 (95% CI 0.78 to 1.68). Compared to the general population, the transplanted patients in the study cohort had a significantly increased malignancy risk (SIR, 2.12; 95% CI 1.01 to 4.44), whereas the nontransplanted patients had no increased risk (SIR, 0.99; 95% CI 0.71 to 1.39).

The SIR calculation for the most common site-specific cancer cases showed significantly increased risks of NMSC (SIR, 3.40; 95% CI 1.62 to 7.14) and hematologic malignancies (SIR, 3.52; 95% CI 1.32 to 9.37). The SIR did not significantly increase in any other site-specific cancer type (Table 2(b)).

### 3.5. Pooled Analysis of the 5 Cohort Studies

**Figure 1:**
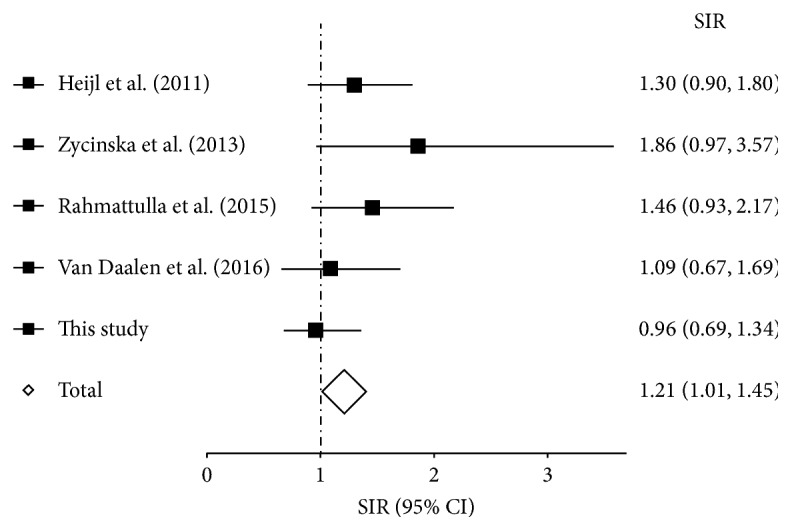
Forest plot showing the risk of malignancy except for nonmelanoma skin cancer in observational studies of patients with ANCA-associated vasculitis. SIR, standardised incidence ratio; 95% CI, 95% confidence interval.

The separate and merged SIRs of all cancer types in the present and the 4 previously reported studies are shown in Table 3 and Figure 1. This analysis included a total of 1532 patients, 8801 patient-years of observation, and 236 cancer cases. The merged SIR of all cancer types was 1.72 (95% CI 1.51 to 1.95) and that of all cancer types, except NMSC, was 1.21 (95% CI 1.01 to 1.45).

## 4. Discussion

In the present study, there was no statistically significant increase in the cancer incidence in the patients with AAV and glomerulonephritis compared to the age- and sex-matched general population (SIR, 1.09; 95% CI 0.89 to 1.49). Excluding the study by Holle et al. that reported an SIR of 0.82 (95% CI 0.45 to 1.38) [25], most previous studies, including those investigating patients with AAV diagnosed after 1988, have reported a significantly increased cancer risk [3–5, 9, 11, 13, 14, 19]. A couple of methodological discrepancies might partially explain the contrasting findings between the present and the majority of previous studies. First, basal skin cell carcinomas are not registered in the Norwegian Cancer Registry and were thus excluded in our analysis. Second, among patients with diagnoses of several cancers, we only included the primary cancer when calculating the SIRs. In contrast, other studies have included subsequent cancer cases, particularly NMSC, in their SIR estimates.

The studies investigating cancer incidences in patients with AAV diagnosed after 1988 reflect contemporary treatment regimens, in which NMSC accounts for the majority of the observed increased malignancy risk [5, 11, 13, 19]. In the present study, the SIR decreased from 1.09 to 0.96 (95% CI 0.69 to 1.34) when NMSC cases were excluded. Further, in the pooled analysis (Table 3), the SIR decreased from 1.72 (95% CI 1.51 to 1.95) to 1.21 (95% CI 1.01 to 1.45) after excluding NMSC. Moreover, part of the residual cancer risk after excluding NMSC can be attributed to posttransplant malignancies, with the SIR posttransplant of 2.12 (95% CI 1.01 to 4.44) in the present study and 4.31 (95% CI 1.17 to 11.04) in that by Van Daalen et al. [19].

In the organ-specific subanalysis, the SIR of NMSC was 3.40 (95% CI 1.62 to 7.14), which is consistent with that of previous studies [5, 9, 11, 13, 14, 19]. An increased risk of NMSC is observed in immunocompromised patients and associated with both environmental factors as chronic human papillomavirus infection [26–29] and a direct effect of individual immunosuppressant, for example, azathioprine [19, 30]. A significantly increased hematologic cancer risk was also found (SIR, 3.52; 95% CI 1.32 to 9.37). Traditionally, CYC use was associated with a very high risk of acute myelogenous leukemia [9, 14, 31]. Interestingly, no case of this cancer form was observed in our cohort, and the increased hematologic malignancy risk was caused by 2 cases of myelodysplastic syndrome and 2 cases of chronic lymphocytic leukemia. To what extent these cases are related to CYC-based therapy or immunosuppression specifically is unclear [32]. In comparison, Zycinska et al. found an increased risk of acute myelogenous leukemia in their study (SIR, 4.3; 95% CI 2.1 to 11.7). Notably, oral CYC was used in the majority of their patients, and 22% received >36 g of cumulative CYC dosage. In addition, a significantly increased urothelial cancer risk was also found in their study, in contrast to those of other recent groups (SIR, 3.4; 95% CI 1.6 to 5.2) [11].

Some previous groups have indicated that the cancer risk, perhaps caused by a higher tendency of relapses and thus higher cumulative immunosuppressive drug doses, is higher in C-ANCA/PR3-ANCA than in P-ANCA/MPO-ANCA positive patients [5, 13]. In contrast, we observed no significantly increased risk in these subgroups compared to the general population.

An important measure to reduce cancer risk in patients with AAV has been replacing CYC with azathioprine for maintenance treatment. This practice change occurred around 2003 concurrent with the publication of the CYCAZAREM study [23, 33]. In the present study cohort, SIR of cancer was not significantly increased, neither in the 1988–2002 nor in the 2003–2012 time periods. Of notice, in a study including a subgroup of the 1988–2002 cohort, cumulative doses of CYC were found fairly low, median 17 grams in intravenous CYC-treated patients [34].

The findings by Van Daalen et al. indicate that rituximab use is associated with a substantially lower risk of malignancy. However, this difference was primarily related to excess NMSC cases. The calculated SIR of cancer, except NMSC, was 1.14 (95% CI 0.49 to 2.25) with CYC and 0.88 (95% CI 0.11 to 3.19) with rituximab treatment. Thus, the SIR of cancer, except NMSC, was only 1.30-fold higher (CI not reported by Van Daalen et al.) in the CYC group than in the rituximab group. Further, the SIR of cancer, except NMSC, was only marginally higher in the present CYC-treated study cohort than in the rituximab group in their study, estimated SIR of 0.96 (95% CI 0.69 to 1.34) and 0.88 (95% CI 0.11 to 3.19), respectively. Finally, no adjustment for posttransplantation malignancies has been performed when comparing CYC- and rituximab-treated patients [19]. In summary, whether replacing low-dose CYC regimens with rituximab would have a beneficial effect on non-NMSC malignancy risk remains uncertain.

NMSC occurrence is still substantially increased in patients with AAV. Although NMSC by no means should be considered as an inconsequential morbidity, deaths caused by these tumors are rare [35]. A number of measures can be taken to reduce the risk and morbidity related to NMSC in immunosuppressed patients; the most important measures include limiting sun exposure of the skin and vigilant monitoring with early NMSC detection and treatment when they appear. Interestingly, Van Daalen et al. observed that rituximab use is associated with a lower NMSC risk than CYC use, which represents a new possible solution to such complication in patients with AAV [19]. However, the decision to replace CYC with rituximab as the first-line treatment in patients with AAV must also include considerations, such as efficacy, treatment-related complications, and cost-benefit.

The major strengths of the present study include its population-based approach and identification of patients with AAV from quality registries with histologic and serologic data. Information on expected and observed cancer cases was retrieved from the same registry, the Norwegian Cancer Registry, which limits potential information biases in the SIR calculation. This registry also has high accuracy and only few missing cases owing to mandatory reporting of cancer cases. Moreover, the pooled analysis strengthens the statistical power and increases the detection rate of significant differences. Some weaknesses of our study must be admitted. Most importantly, we could not correlate our findings to the cumulative CYC doses administered. Treatment data were unavailable in the NKBR. However, we have shown from previous reports of this cohort that these patients have received therapy according to international recommendations [22–24]. Another weakness is the lack of information regarding extrarenal and relapsing disease. Relapsing disease in particular is associated with increased treatment length and cumulative doses of immunosuppressive drugs that might affect risk of cancer development. Also, owing to a lack of sufficient data, we could not calculate pooled SIRs for single-site cancers or for malignancy after excluding posttransplant cancers.

In summary, we have demonstrated that the risk of malignancy in patients with AAV and glomerulonephritis is not significantly increased. However, significantly increased NMSC, hematologic malignancy, and posttransplantation cancer risks were found. These findings indicate the presence of associations, although relatively weak, between immunosuppression and cancer development. However, recently published data suggest that substituting CYC with rituximab could eliminate the risk of developing NMSC. Our findings confirm that the long-term international efforts of developing CYC-minimizing strategies had an important beneficial effect on cancer morbidity in patients with AAV.

## Figures and Tables

**Table 1:** Baseline demographics of 419 Norwegian patients with AAV.

Characteristic	Total	Nonmalignancy	Malignancy	*p* value
Age (median, IQR)	62 (49–72)	61 (48–72)	65 (56–73)	0.04
Male sex (%)	229 (55%)	200 (53%)	29 (71%)	0.03
eGFR (median, IQR)	23 (11–46)	24 (11–47)	19 (9–39)	0.29
C-ANCA/PR3-ANCA	237 (57%)	213 (56%)	24 (59%)	0.89

AAV, ANCA-associated vasculitis; IQR, interquartile range; eGFR, estimated glomerular filtration rate, mL/min/1.73 m^2^; C-ANCA, cytoplasmic ANCA; PR3-ANCA, proteinase 3 ANCA.

**Table:** (a) Standardised incidence ratios for cancers in all sites in the study population

Characteristic	Observed	Expected	SIR	95% CI
All	41	37.5	1.09	0.81 to 1.49
Non-NMSC	34	35.4	0.96	0.69 to 1.34
Sex				
Male	29	22.9	1.27	0.88 to 1.83
Female	12	14.6	0.82	0.47 to 1.44
Follow-up period				
0-1 year	3	4.3	0.70	0.22 to 2.16
1–5 years	22	14.4	1.53	1.01 to 2.32
5–10 years	11	11.1	0.99	0.55 to 1.78
>10 years	5	7.6	0.66	0.27 to 1.57
Transplantation				
Yes	7	3.3	2.12	1.01 to 4.44
No	34	34.2	0.99	0.71 to 1.39
ANCA serology				
C-ANCA/PR3-ANCA	24	20.6	1.17	0.78 to 1.74
P-ANCA/MPO-ANCA	17	16.9	1.01	0.63 to 1.62
Study period				
1988–2002	26	22.7	1.14	0.78–1.68
2003–2012	15	14.8	1.02	0.61–1.68

SIR, standardised incidence ratio; 95% CI, 95% confidence interval; NMSC, nonmelanoma skin cancer; C-ANCA, cytoplasmic ANCA; PR3-ANCA, proteinase 3 ANCA; P-ANCA, perinuclear ANCA; MPO-ANCA, myeloperoxidase ANCA.

**Table:** (b) Standardised incidence ratios for the most common organ-specific cancers in the study population

Organs	Observed	Expected	SIR	95% CI
NMSC	7	2.1	3.40	1.62 to 7.14
Hematologic	4	1.1	3.52	1.32 to 9.37
Lung	7	4.0	1.73	0.83 to 3.63
Colon	2	1.2	1.73	0.43 to 6.93
Urothelium	3	2.0	1.48	0.47 to 4.59
Prostate	5	7.0	0.72	0.30 to 1.73
NHL	1	1.2	0.86	0.12 to 6.12

SIR, standardised incidence ratio; 95% CI, 95% confidence interval; NMSC, nonmelanoma skin cancer; NHL, non-Hodgkin lymphoma.

**Table 3:** Studies on cancer incidence in patients with AAV.

Characteristic	Heijl et al. (2011)	Zycinska et al. (2013)	Rahmattulla et al. (2015)	Van Daalen et al. (2016)	This study	Total
Study period	1995–2007	1990–2008	1991–2013	2000–2014	1988–2012	
Number of patients	535	117	138	323	419	1532
Cumulative person-years	2650	NR	1339	1802	3010	8801
Number of observed cancers	50	15	85	45	41	236
Number of expected cancers	31.7	6^a^	38.5^a^	23.8	37.5	137.5
SIR (95% CI)	1.58 (1.17 to 2.08)	2.50 (1.20 to 2.90)	2.21 (1.64 to 2.92)	1.89 (1.38 to 2.53)	1.09 (0.81 to 1.49)	1.72 (1.51 to 1.95)

*All non-NMSC sites *						
Number of observed cancers	35	9^a^	24	20	34	122
Number of expected cancers	25.9	4.8^a^	16.4^a^	18.33	35.4	100.8
SIR (95% CI)	1.30 (0.90 to 1.80)	1.86^a^ (0.97 to 3.57)	1.46 (0.93 to 2.17)	1.09 (0.67 to 1.69)	0.96 (0.69 to 1.34)	1.21 (1.01 to 1.45)

^a^Not reported; calculated by the authors of this study; SIR, standardised incidence ratio; 95% CI, 95% confidence interval; NR, not reported; NMSC, nonmelanoma skin cancer.
